# High Levels of SOX5 Decrease Proliferative Capacity of Human B Cells, but Permit Plasmablast Differentiation

**DOI:** 10.1371/journal.pone.0100328

**Published:** 2014-06-19

**Authors:** Mirzokhid Rakhmanov, Heiko Sic, Anne-Kathrin Kienzler, Beate Fischer, Marta Rizzi, Maximilian Seidl, Kerstina Melkaoui, Susanne Unger, Luisa Moehle, Nadine E. Schmit, Sachin D. Deshmukh, Cemil Korcan Ayata, Wolfgang Schuh, Zhibing Zhang, François-Loic Cosset, Els Verhoeyen, Hans-Hartmut Peter, Reinhard E. Voll, Ulrich Salzer, Hermann Eibel, Klaus Warnatz

**Affiliations:** 1 Center for Chronic Immunodeficiency (CCI), University Medical Center and University Freiburg, Freiburg, Germany; 2 Department of Pneumology, University Medical Center and University Freiburg, Freiburg, Germany; 3 Division of Molecular Immunology, Department of Internal Medicine III, Nikolaus Fiebiger Center, University of Erlangen-Nürnberg, Erlangen, Germany; 4 Department of Obstetrics and Gynecology, Virginia Commonwealth University, Richmond, Virginia, United States of America; 5 CIRI, INSERM U1111, EVIR, Ecole Normale Supérieure de Lyon, Université de Lyon, Lyon, France; 6 INSERM U1065, C3M, Metabolic control of cell deaths, Nice, France; 7 Division of Rheumatology and Clinical Immunology, University Medical Center Freiburg, Freiburg, Germany; DRFZ, Germany

## Abstract

Currently very little is known about the differential expression and function of the transcription factor SOX5 during B cell maturation. We identified two new splice variants of *SOX5* in human B cells, encoding the known L-SOX5B isoform and a new shorter isoform L-SOX5F. The *SOX5* transcripts are highly expressed during late stages of B-cell differentiation, including atypical memory B cells, activated CD21^low^ B cells and germinal center B cells of tonsils. In tonsillar sections SOX5 expression was predominantly polarized to centrocytes within the light zone. After *in vitro* stimulation, *SOX5* expression was down-regulated during proliferation while high expression levels were permissible for plasmablast differentiation. Overexpression of L-SOX5F in human primary B lymphocytes resulted in reduced proliferation, less survival of CD138^neg^ B cells, but comparable numbers of CD138^+^CD38^hi^ plasmablasts compared to control cells. Thus, our findings describe for the first time a functional role of SOX5 during late B cell development reducing the proliferative capacity and thus potentially affecting the differentiation of B cells during the germinal center response.

## Introduction

Sox (sex determining region Y (SRY)-related high-mobility-group (HMG)-box) family of proteins are encoded by 20 genes in humans and mice and are classified into eight groups - group SoxA to SoxH - according to the sequence identity in their DNA-binding HMG-domain and other conserved regions (reviewed in [1], [2]). Sox proteins function as transcription factors and play important roles in many developmental and cellular processes. Although most Sox proteins predominantly serve as transcriptional activators, there is also evidence for transcriptional repression and architectural roles (reviewed in [3]). Essential roles and key functions in cell fate decisions have been identified for Sox proteins in sex differentiation, neurogenesis and gliogenesis, neural crest development, skeletogenesis, cardiogenesis and angiogenesis as well as in hematopoiesis [1], [3].

Sox5 belongs to the SoxD group composed of *Sox5*, *Sox6* and *Sox13*, in vertebrates (reviewed in [4]). SoxD group genes and protein structures are highly conserved in the family-specific HMG-domain and in the group-specific leucine zipper and coiled-coil domains [3], [4]. The SoxD HMG-box domain preferentially binds the DNA consensus sequence of AACAAT [5] and SoxD proteins can act as either transcriptional activators or repressors [4]. Each *SoxD* gene is expressed in a limited subset of cell types [4]. High levels of *Sox5* and *Sox6* gene co-expression are found in spermatids, neurons, oligodendrocytes and chondrocytes [6]–[9].

The human SOX5 protein exists in a short (S-SOX5) and long (L-SOX5) isoform, encoded by a unique transcript for S-SOX5 and by several transcript variants for L-SOX5 isoforms. While in humans the short isoform is expressed mainly in the testes [10], high levels of long isoforms are found in fetal brain [10], striated muscles and chondrocytes [11]. Knock-out mouse models demonstrated crucial roles of L-SOX5 in developmental and cellular processes during chondrogenesis [12] and neurogenesis [13], [14], but very little is known about its expression and function in B lymphocytes. Comparative transcriptome analysis of different memory B-cell subpopulations from healthy donor (HD) tonsils revealed differential regulation of *SOX5*, along with other markers like *RUNX2*, *DLL1* and *AICDA*, as a distinctive signature profile of FCRL4^+^ memory B cells [15]. Later, dysregulation of *SOX5* gene expression was reported in the innate-like CD21^low^ B-cell subpopulation of patients with common variable immunodeficiency (CVID) [16] and patients with hepatitis C virus-associated mixed cryoglobulinemia [17]. Attempts to test the function of SOX5 in the activation of *FCRL4* promoters did not reveal any significant influence of SOX5 in the regulation of the *FCRL4* gene expression [15]. Since the function of SOX5 in B cells still remains elusive, we aimed in this study to investigate the expression and function of SOX5 in human B cells. We describe the differential expression of *SOX5* transcripts during B cell development. Combined with functional assays *in vitro* these findings expose a new role and function of SOX5 in human terminal B cell differentiation.

## Materials and Methods

### HD Individuals’ Material

The study was approved by the internal ethics board (University Hospital Freiburg 313/04 and 121/11).Iinformed written consent was obtained from each individual before participation in the study, in accordance with the Declaration of Helsinki.

### B Cell Isolation and In vitro Stimulation

B cells were isolated by negative magnetic bead selection using the MACS B Cell Isolation Kit II (Miltenyi Biotec) according to manufacturer’s instructions. The purity of >95% was reached in B cell fractions. The cells were stimulated *in vitro* for 9 days at 37°C in RPMI 1640 medium containing 10% FCS either in the presence of IL4, IL21, CD40L or a combination of IL4+ CD40L +/− IL21. IL4 (ImmunoTools) was used at the final concentration of 100 U/ml. Preparation of CD40L and IL21 was previously described [18]. Prior to use CD40L and IL21 containing supernatants were concentrated and titrated.

### Preparation of Tonsillar B Cells

Tonsillar single cell suspensions were prepared by tissue mincing, filtration through 70-µm nylon filters and centrifugation on a Ficoll gradient. The cells were stained with appropriate antibodies and subjected to cell sorting.

### Flow Cytometry and Cell Sorting

The following antibodies were used: FITC-anti-CD38 (BD Pharmingen), PE-anti-IgD (Southern Biotechnology Associates, Inc.), PE-anti-CD138 (Coulter-Immunotech), PerCP-Cy5.5-anti-CD27 (Biolegend), PE-Cy7-anti-CD21 (clone B-ly4, BD Pharmingen), PE-Cy7-anti-CD3 (Beckman Coulter), Cy5-anti-IgM (Jackson ImmunoResearch Laboratories, Inc.) and APC-H7-anti-CD19 (clone SJ25C1, BD Biosciences).

FACS CantoII and LSR II (BD Biosciences) cytometers were used to perform flow cytometric analysis. FACS data were analyzed using FlowJo (Tree Star Inc.) software.

For cell sorting PBMCs were sorted into CD19^+^IgM^+^CD21^+^CD38^+^CD27^−^ naïve B cells, CD19^+^IgM^hi^CD21^+^ CD38^+^CD27^+^ MZ-like B cells, CD19^+^IgM^−^CD21^+^CD38^+^CD27^+^ switched memory B cells, CD19^+^IgM^−^CD21^+^CD38^+^CD27^−^ non-classical memory B cells and CD19^+^IgM^+/−^CD21^low^ CD38^low^ B cells, as well as tonsillar B cells into CD19^+^IgD^+^CD27^−^CD38^−^ follicular naive B cells, CD19^+^IgD^−^CD27^−/+^CD38^+^ GC B cells, CD19^+^IgD^−^CD27^++^CD38^++^CD138^+^ plasma cells (PC) and CD19^+^IgD^−^CD27^+^CD38^−^ memory B cell populations to a purity of >95% using a MoFlow cell sorter (Beckman Coulter).

### RT-PCR and qPCR Assays

Total RNA was extracted using TRIzol reagent (Life Technologies) and on column DNase digestion was performed during purification of total RNA using PureLink RNA Mini columns (Life Technologies) according to manufacturer’s instructions. Subsequently, cDNA was prepared by reverse transcription using oligo-(dT)_20_ primer (Life Technologies) and SuperScript II Reverse Transcriptase (Life Technologies) for further PCR analysis.

qPCR for *SOX5*, *BLIMP1* and ribosomal protein, large, P0 (*RPLP0*) genes was performed by a LightCycler 480 Real-Time PCR System (Roche Diagnostics GmbH) and based on SYBR Green I detection (LightCycler 480 SYBR Green I Master mix, Roche Diagnostics GmbH) using the following conditions: activation at 95°C for 5 min and 50 cycles of amplification at 95°C for 10 s, 63°C for 20 s and 72°C for 30 s., followed by subsequent melting curve analysis. *RPLP0* gene was used as an endogenous internal control in samples. PCR primer sequences are provided in the table S1.

### Human Cell Cycle RT^2^ Profiler PCR Array Assays

Human cell cycle RT^2^ Profiler PCR array (Qiagen) assays were run in samples of GFP- and SOX5-GFP-transduced human primary B cells at day 3 upon CD40L+IL4+IL21 stimulation *in vitro*. 400 ng of total RNA was used for each array and the assays were performed according to the manufacturer’s instructions.

### PCR Cloning and DNA Sequencing

The complete coding sequence (CDS) of *SOX5* gene was amplified from a HD B cell cDNA sample as a 3.1 kb PCR fragment using proof-reading Phusion DNA polymerase (New England BioLabs). PCR products were subcloned into the pCRII TOPO TA vector (Life Technologies) according to manufacturer’s instructions.

DNA sequencing was performed by an ABI Prism 377 DNA Sequencer (PE, Applied Biosystems). Sequencing data were analyzed using the DNA Sequencing Analysis software version 3.4 (PE Applied Biosystems) and Sequencer version 3.4.1 (Gene Codes Corporation). Assembly, alignment and analysis of DNA sequences were performed by DNASTAR Lasergene software version 8.0 (DNASTAR, Inc.). Cloning and sequencing primers are provided in the table S1.

### Fragment Analysis Assay

*SOX5* transcript variant 6 (5′L3L**-** *SOX5*) and variant 7 (5′L3S**-** *SOX5*) were amplified in one reaction using 5′-FAM labeled primers and subsequent fragment analysis was performed using the 3130xl Genetic Analyzer (Applied Biosystems) according to the manufacturer’s instructions. GeneScan–500 LIZ (Applied Biosystems) was used as a size standard in the assays.

### Promoter Reporter Assays

The promoter activity was assessed using BEAS-2B human bronchial epithelial cells and human *SPAG6* gene promoter reporter constructs, as previously described [19]. The promoter activity was measured with the Dual Luciferase Reporter Assay system (Promega) according to manufacturer’s instructions.

### Immunofluorescence Staining and Microscopy

Cyto-spins of RAJI cells were fixed for 15 min using 4% solution of freshly prepared formaldehyde in PBS and permeabilized with 0.5% Triton-X for another 15 min. After rinsing and blocking using 3% BSA in PBS the cells were stained with rabbit anti-human SOX5 antibodies (Santa Cruz Biotechnolgies, Inc.) over night at 4°C in the blocking buffer, followed by staining with TRITC-conjugated swine anti-rabbit IgG antibodies (DakoCytomation) for 1 h at room temperature (RT). After appropriate washing steps DAPI was applied on slides for 10 min to stain nuclei.

Confocal microscopy images were collected using the LSM 710 Laser Scanning Microscope and the Image software ZEN black 2011 (Carl Zeiss) with following settings: 100X magnification- Plan-Apochromat 100X/1.40 oil differential interference contrast objective lens, 266 mm numerical aperture/pinhole, static sample with respect to temperature and DAKO mounting medium. Images were presented using Photoshop CS5 gamma 0.45.

Cryosections (10 µm) of tonsillar tissues were fixed with acetone and blocked using 5% goat serum (Vector Labs) in PBS for 1.5 h at RT. Primary (goat anti-human IgD-bio (Southern Biotech.); rabbit anti-human SOX5 (Santa Cruz Biotechnolgies, Inc.); mouse anti-human Ki-67 (BD) and mouse anti-human CD138 (Dako) antibodies) and secondary antibody (goat anti-mouse IgG-A405 (Invitrogen); goat anti-rabbit IgG-A568 (Invitrogen) and SA-FITC (Dako)) staining was performed in the blocking buffer containing 5% goat serum for 1 h at RT. Microscopic images were acquired using Axio Observer and AxioCam MRm (Zeiss) and were prepared using Axiovision (Ver. 4.8.2.0) and Photoshop CS5 softwares.

### Engineering of the SOX5-GFP Fusion Protein Construct and Lentiviral Transduction

A 2.4 kb complete CDS fragment of the *5′L3S* **-** *SOX5* transcript was cloned into the pNL-EGFP/CEF lentiviral vector [20], [21] and a C-terminus-GFP fusion construct – pNL-L-SOX5F-EGFP/CEF, expressing L-SOX5F-EGFP fusion protein, was generated using EcoRI and BamHI cloning sites. In the following sections the pNL-EGFP/CEF control vector and pNL-L-SOX5F-EGFP/CEF fusion constructs were designated as GFP and SOX5-GFP, respectively. Lentiviral particles were prepared by co-transfection of pLTR-G envelop [22] and pCD/NL-BH* helper [23] constructs in 293T cells, as a packaging cell line, using FuGENE 6 (Roche Diagnostics). Viral particles were harvested after 48 h and after two rounds of spin infection RAJI cells were GFP sorted for further analysis. Cloning primers are provided in the table S1.

Isolated quiescent human peripheral blood primary B cells were transduced by novel lentiviral vectors (LVs) displaying at their surface the Edmonston measles virus (MV) glycoproteins hemagglutinin (H) and fusion protein (F), (H/F-LVs) technique [24], [25]. In order to transduce primary B cells, H/F-LV vector pseudotypes were prepared for the SOX5-GFP fusion as well as for GFP encoding vector, as a control and the cells were transduced using the above viral particles. The efficiency of transduction up to 85.0% was reached in SOX5-GFP H/F-LV transduced B cells and it was even higher in the case of GFP control H/F-LVs (up to 99.0%) after two rounds of spinoculation/infection.

### Statistical Analysis

All data are expressed as mean ± standard deviation (± SD). Statistical comparison between samples was carried out by Kruskal-Wallis (KW) one way analysis of variance (ANOVA) test and in cases of significant differences, t-test was applied to indicate the significance between two groups of samples using GraphPad Prism 5 software (GraphPad Software, La Jolla, CA).

## Results

### Expression of SOX5 Transcript Variants in Human B Cells

**Figure 1:**
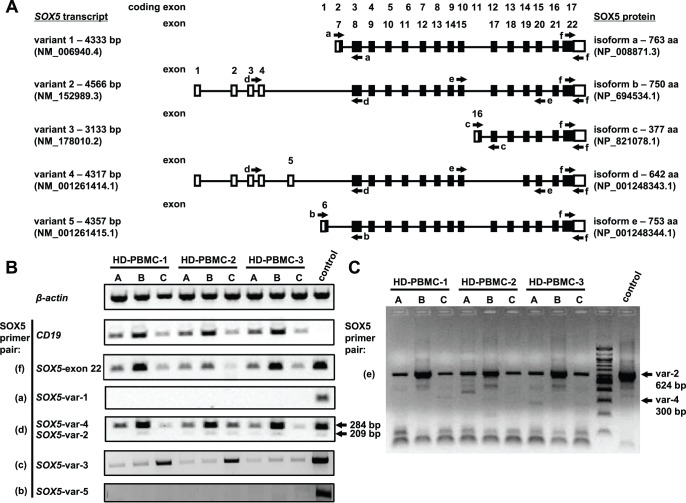
Expression of *SOX5* transcript variants in human B cells. (A) Schematic representation of human *SOX5* transcript variants. Non-coding exons are depicted as open rectangles, partial coding exons - as half open rectangles and coding exons - as filled rectangles. Primer regions are indicated with appropriate arrows. Exons and coding exons are numbered according to their location along the genomic sequence, which are drawn as black lines. (B) RT-PCR analysis for the expression of *β-actin*, *CD19* genes and *SOX5* transcript variants. HD PBMCs were separated into: A – PBMCs; B – B cells and C – non-B lymphocytes. Except for *SOX5* transcript variant 3 (*SOX5*-var 3) in which human testis RNA sample served as a control, human costal cartilage cells used as a positive control in all RT-PCR reactions. In agarose gel pictures DNA markers were cut out, since they were loaded between the tested samples and the control sample. (C) RT-PCR assay performed to discriminate between *SOX5* transcript variant 2 and variant 4 in samples of peripheral blood lymphocytes: A – PBMCs; B – B cells and C – non-B lymphocytes.

*SOX5* transcript variants are encoded in total by 22 exons, out of which 17 are coding exons (Fig. 1A). In order to analyze the expression of *SOX5* transcripts in B cells, cDNA was prepared from three different fractions of peripheral blood mononuclear cells (PBMCs) of three HD volunteers: fraction A – original pool of PBMCs, fraction B – “untouched” B cells and fraction C – non-B cells (Fig. 1B). PCR analyses did not reveal detectable signals for *SOX5* transcript variant 1 (primer pair a) and 5 (primer pair b) in human B cells or other peripheral blood lymphocytes, while the signal was readily detectable in human costal cartilage cells (Fig. 1B). B cells expressed low levels of *SOX5* variant 3 (primer pair c) compared to the non-B cell fraction and human testis tissue (Fig. 1B). A weak signal at about expected 209 bp - corresponding to variant 2 - and a strong signal at the expected 284 bp - corresponding to variant 4 - were detected in B cells by primer pair d (Fig. 1B). Since the presence or absence of exons 17 and 18 is an additional difference between *SOX5* transcript variants 2 and 4, a new set of primer pair (in exons 15 and 20, primer pair e) was designed discriminating between these two variants (Fig. 1A). Surprisingly, the results revealed no detectable signal at 300 bp corresponding to the variant 4, but a strong signal at the expected 624 bp fragment corresponding to the *SOX5* transcript variant 2 (Fig. 1C). These data indicated that *SOX5* transcript variant 4 is not expressed in B cells and suggested that the observed 209 bp and 284 bp fragments correspond to sub-variants of *SOX5* transcript variant 2. Overall, B cells were the main source among human peripheral blood lymphocytes, expressing high levels of long transcript variants of *SOX5* (primer pairs – d, e and f; Fig. 1B and 1C).

### Human B Cells Express at Least 3 Different L-SOX5 Transcript Variants

**Figure 2:**
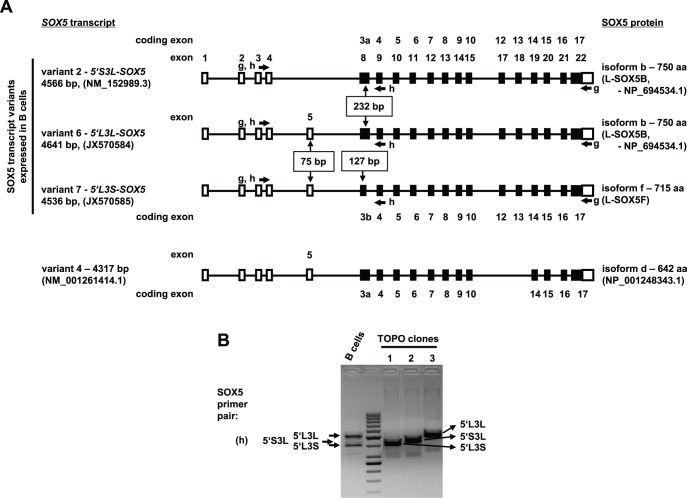
Human B cells express at least three different transcript variants of *SOX5*. (A) Schematic representation of sequence verified human *SOX5* transcript variants in B lymphocytes. Non-coding exons are depicted as open rectangles, partial coding exons - as half open rectangles and coding exons - as filled rectangles. Cloning primer locations are indicated with appropriate arrows. Exons and coding exons are numbered according to their location along the genomic sequence, which are drawn as black lines. (B) PCR analysis of *SOX5* transcript expression in B cells and in single clones picked for sequence analysis. B lymphocytes express at least three different *SOX5* transcript splice variants as evidenced by representative single TOPO clones 1, 2 and 3.

Due to the predominance of *SOX5* transcript variant 2, we decided to amplify the complete coding sequence (CDS) of the *SOX5* transcript variant 2 (primer pair g) from human peripheral blood B lymphocytes, in order to subclone the PCR fragments and verify the sequence of B cell-specific *SOX5* transcripts. Sequencing indeed revealed a transcript corresponding to the *SOX5* transcript variant 2 (NM_152989.3) encoding the L-SOX5B isoform (NP_694534.1). Additionally, we identified two new *SOX5* transcript variants and numbered them accordingly as variant 6 (GenBank accession number: JX570584) and variant 7 (GenBank accession number: JX570585), both of which contained the 75 bp non-coding exon 5 at the 5′-UTR known from variant 4, but in contrast to variant 4 and similar to variant 2, variant 6 and 7 enclose coding exons 12 and 13 (Fig. 2A). The coding exon 3 was present in its full length of 232 bp (coding exon 3a) in *SOX5* transcript variant 6 and it was 105 bp shorter due to a truncated 127-bp coding sequence (coding exon 3b) in transcript variant 7 (Fig. 2A). Therefore, the three *SOX5* transcripts were designated as *5′S3L*-*SOX5*, corresponding to the known transcript variant 2 (NM_152989.3), as well as *5′L3L*-*SOX5* (transcript variant 6) and *5′L3S*-*SOX5* (transcript variant 7), representing two additional new transcript variants of *SOX5*, where 5′S describes the existence of shorter and 5′L - of longer 5′-UTR sequences, whereas 3L indicates the longer and 3S - the shorter coding exon 3, respectively. According to the analysis of open reading frames, both transcript variant 2 (*5′S3L*-*SOX5,* NM_152989.3) and variant 6 (*5′L3L*-*SOX5,* JX570584) encode the same protein isoform, i.e. the 750 amino acid (aa) long L-SOX5B protein (NP_694534.1). The transcript variant 7 (*5′L3S*-*SOX5,* JX570585) encodes a new, 35 aa shorter form of L-SOX5, due to the 105 bp shorter version of coding exon 3b (Fig. 2A). We named the new isoform L-SOX5F (GenBank accession number to be assigned).

As expected, a PCR assay recognizing all *SOX5* transcript variants in one reaction (primer pair h) revealed the existence of at least three different transcript variants, which were confirmed by single clone sequencing in human B cells (Fig. 2B). Intriguingly, in B lymphocytes the 5′L- transcript variants of *SOX5*, containing the 75-bp-non-coding exon 5, were more abundant compared to the 5′S- transcript variant of *SOX5* (Fig. 1B and Fig. 2B).

### Expression of SOX5 in Human B Cell Populations

**Figure 3:**
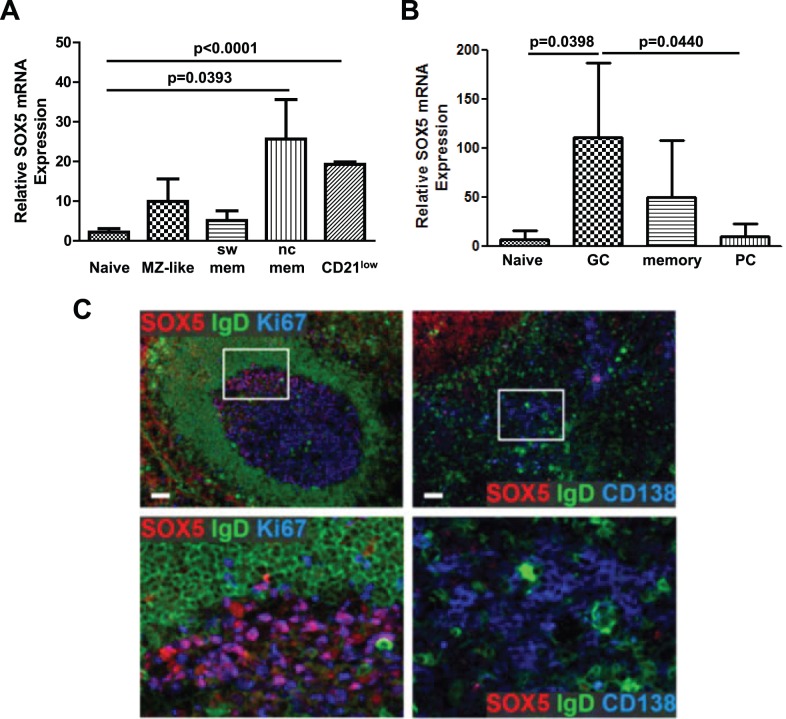
Expression of *SOX5* in human B cell subpopulations. (A) Relative quantification of *SOX5* by RT-qPCR in peripheral blood naive, MZ-like, switched memory (sw mem), non-classical memory (nc mem) and CD21^low^ B cells. (B) Relative quantification RT-qPCR assay for *SOX5* expression in follicular naive, germinal center B cells (GC), memory B cells and plasma cells (PC) from tonsils. T-test p-values indicate the significance of differences between the samples. Relative expression levels of *SOX5* are shown as mean ± SD. *RPLP0* gene was used as an internal control in the samples. (C) Immunofluorescence staining for the expression of SOX5 protein in tonsillar tissues. IgD staining was used to stain mantle zones, Ki67 staining for proliferating centroblasts within germinal centers and CD138 as a marker for extrafollicular plasma cells.

Next, we analyzed *SOX5* expression in circulating naive CD19^+^IgM^+^CD21^+^CD38^+^CD27^−^ B cells, CD19^+^IgM^hi^CD21^+^CD38^+^CD27^+^ marginal zone (MZ)-like B cells, CD19^+^IgM^−^CD21^+^CD38^+^CD27^+^ switched memory B cells, CD19^+^IgM^−^CD21^+^CD38^+^CD27^−^ non-classical memory B cells and CD19^+^IgM^+/−^CD21^low^CD38^low^ innate-like CD21^low^ B cells. Quantitative RT-PCR analysis, recognizing all transcript variants of SOX5 expressed in B cells (primer pair – f), showed that expression levels of *SOX5* transcripts were slightly, but not significantly higher in MZ-like and switched memory B cells compared to naive B cells. In contrast, levels of *SOX5* transcripts were 25.7±17.2 fold (n = 4; p = 0.0393) and 19.3±1.2 fold (n = 4; p<0.0001) higher in non-classical memory and CD21^low^ B cells, respectively, than in naive B cells (Fig. 3A).

In addition to circulating B cells we examined tonsillar B cell subsets sorted into CD19^+^IgD^+^CD27^−^CD38^−^ follicular naive B cells, CD19^+^IgD^−^CD27^−/+^CD38^+^ germinal center (GC) B cells, CD19^+^IgD^−^CD27^++^CD38^++^CD138^+^ plasma cells (PCs) and CD19^+^IgD^−^CD27^+^CD38^−^ memory B cells. Compared to follicular naive B cells GC B cells expressed 110.5±76.4 fold (n = 3; p 0.0398) higher levels of *SOX5* transcripts (Fig. 3B). The expression levels of *SOX5* transcripts were not significantly higher in memory B cells, whereas PCs expressed significantly (p = 0.0440) lower levels of *SOX5* transcripts compared to the GC B cells. Consistently, immunofluorescence staining of tonsillar tissues revealed polarized expression of SOX5 protein preferentially in centrocytes within the light zone of germinal centers, but not in terminally differentiated CD138^hi^ extrafollicular plasma cells (Fig. 3C).

In order to distinguish between the two prominent SOX5 transcript variants we designed a qPCR assay recognizing only the transcript variant 6 (*5′L3L*-*SOX5*, Fig. S1A, primer pair – i) in B cells. Except for the non-classical memory B cells in the blood, transcript varrinat 6 (5′L3L-SOX5) expression reflected (Fig. S1B and S1C) the expression pattern of total SOX5 variants in peripheral blood and tonsillar naïve, GC and memory B cells (Fig. 3A and 3B). Subsequent fragment analysis using 5′-FAM-labeled primers (Fig. S1A, primer pair - j) revealed a nearly equal distribution between transcript variant 7 (*5′L3S*-*SOX5)* and variant 6 (*5′L3L*-*SOX5*) in all tested tonsillar B cell populations (Fig. S1D). While the fragment analysis was not sensitive enough to analyse peripheral blood B cells *ex vivo*, the analysis of B cells stimulated *in vitro* (Fig. S1E) showed a similar expression pattern of these variants. Collectively, these data demonstrate that both transcript variant 6 (*5′L3L*-*SOX5*) and variant 7 (*5′L3S*-*SOX5*) are equally expressed in B cells *ex vivo* as well as in B cells during activation *in vitro*.

### SOX5 Expression during Activation and Differentiation of B Cells In vitro

**Figure 4:**
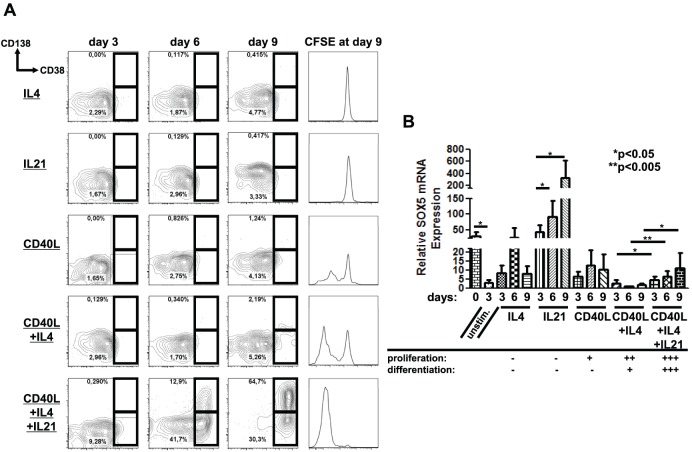
Induction of *SOX5* during *in vitro* B cell differentiation. (A) Isolated peripheral blood B cells were either left for 3 days without any stimulus or stimulated for 9 days *in vitro* either with a single stimulus (IL4, IL21 and CD40L) or the combination of these (CD40L+IL4+/− IL21). The cells were analyzed by FACS for the plasma cell markers CD138 and CD38 at days 3, 6 and 9. Gates indicate the frequency of CD138^+^CD38^hi^ plasmablasts in each plot. CFSE at day 9 was measured as an indicator of proliferation in the cells. Representative FACS plots of five independent experiments are shown. (B) RT-qPCR analysis of *SOX5* expression in ex vivo B cells and samples either unstimulated (unstim.) for 3 days or stimulated with a single stimulus (IL4, IL21 and CD40L) or the combination of these (CD40L+IL4+/− IL21). Significant differences are depicted and appropriate t-test p-values (n = 5; p<0.05) are indicated. Relative expression levels of *SOX5* are shown as mean ± SD. *RPLP0* gene served as an internal control in the samples.

Since high levels of *SOX5* mRNA were found at some activation dependent stages of B cell differentiation in the tonsil, we asked next whether specific signaling pathways might induce SOX5 expression during *in vitro* stimulation of B cells. Isolated peripheral blood B cells were activated either with a single stimulus (IL4, IL21 and CD40L) or combinations of stimuli (CD40L+IL4 and CD40L+IL4+IL21) *in vitro*. Flow cytometric analysis at days 3, 6 and 9 revealed strong differentiation of CD38^hi^CD138^+^ plasmablasts upon CD40L+IL4+IL21 stimulation and only a weak differentiation upon CD40L+IL4 stimulation, whereas no differentiation was detectable in cells activated with IL4 or IL21 or CD40L alone (Fig. 4A). Proliferation, measured by CFSE labeling of cells, occurred to a small degree upon CD40L stimulation, stronger in cells stimulated with CD40L+IL4 and the strongest after CD40L+IL4+IL21 stimulation (Fig. 4A), while B cells stimulated with IL4 and IL21 alone did not proliferate. We noticed a significant decrease of *SOX5* expression (p<0.05) in unstimulated B cells at day 3 compared to freshly isolated B cells (Fig. 4B). Subsequently, *SOX5* transcripts were highly up-regulated upon stimulation with IL21 (Fig. 4B). In contrast, all proliferating cells after IL4+CD40L stimulation expressed the lowest amounts of *SOX5,* which was slightly but significantly increased by the addition of IL-21 (n = 5, p-values<0.05) (Fig. 4B). These data suggested a reduction of *SOX5* expression during proliferation, while higher levels of SOX5 were present in culture conditions inducing plasmablast differentiation. In order to test whether *SOX5* is induced not only during IL21-mediated differentiation, isolated peripheral B cells were stimulated with CpG *in vitro*. Upon CpG stimulation, CD38^hi^CD138^+^ plasmablast differentiation was observed at days 6 and 9 (Fig. S2A) and this process was also associated with increased levels of *SOX5* transcripts in the cells (Fig. S2B).

### Expression of SOX-trio and SOX13 Genes in Human Ex vivo B Cells and during In vitro B Cell Differentiation

**Figure 5:**
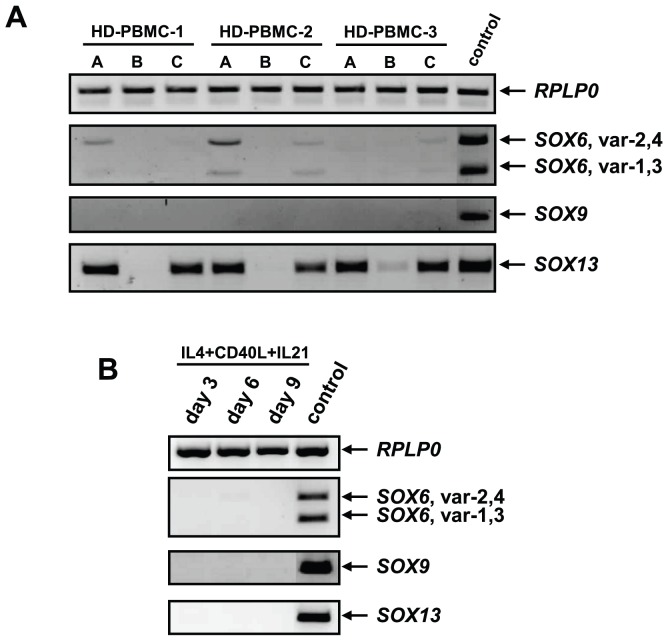
Expression of “SOX-trio” and *SOX13* genes in *ex vivo* B cells and during *in vitro* differentiation of human primary B cells. (A) PCR analysis for the expression of *RPLP0*, *SOX6*, *SOX9* and *SOX13* genes in human peripheral blood lymphocyte populations: A – PBMCs; B – B cells and C – non-B cell fractions. (B) *RPLP0*, *SOX6*, *SOX9* and *SOX13* expression in isolated peripheral blood B cells stimulated with IL4+CD40L+IL21 for 9 days *in vitro*. The analysis was performed at days 3, 6 and 9. In agarose gel pictures DNA markers were cut out, since they were loaded between the tested samples and the control sample. Human costal cartilage cells served as a control for *SOX6* and *SOX9* expression, whereas BEAS-2B cells were used for *SOX13* as a control.

Since *Sox5* is co-expressed with *Sox6* and *Sox9* in chondrocytes, oligodendrocytes and melanocytes [4], we asked whether the other two members of the SOX-trio [26] are expressed in human B cells. Human *SOX9* gene is represented by a single transcript, while at least four different transcript variants exist for the human *SOX6* gene. PCRs for *SOX9* and *SOX6* transcripts, recognizing all four transcript variants of *SOX6*, showed weak signals for *SOX6* transcript variants in PBMCs and non-B cell fractions, but were absent in B cells (Fig. 5A). *SOX9* was undetectable in any lymphocyte population of the peripheral blood, while the signal was readily detected in human costal cartilage cells (Fig. 5A).

Since co-expression of *Sox5* and *Sox13* genes was reported in mouse pancreatic epithelial cells [27], we tested co-expression of the *SOX13* gene in human B lymphocytes. While SOX13 was expressed in non-B cell fractions of the PBMC, there was no detectable signal in B cell fractions, indicating that *SOX13* is not expressed in human B cells (Fig. 5A).

Because freshly isolated B cells did not express *SOX6* and *SOX9* or *SOX13 ex vivo*, we tested whether these genes are induced during *in vitro* B cell differentiation. As shown in Fig. 5B, none of the tested genes were induced at days 3, 6 or 9 after IL4+CD40L+IL21 stimulation.

### SOX5 Overexpression Reduces Proliferation, While Plasmablast Differentiation Still Occurs

Because of its novelty and the prominent expression in B cells, we decided to clone the transcript variant 7 of *SOX5* (*5′L3S*-*SOX5)* in order to study its function in human B cells. A SOX5-GFP fusion protein was engineered and its functionality was tested by using the previously reported *SPAG6* promoter reporter assays. In BEAS-2B epithelial cells expressing the SOX5-GFP fusion protein, the *SPAG6* promoter activities reached much higher induction levels, 14.2±1.2 fold for pGL3-1-SOX5 (n = 3; p = 0.0005) and 10.2±0.6 fold for pGL3-4-SOX5 (n = 3; p<0.0001) when compared with controls (Fig. S3A), indicating that our SOX5-GFP fusion protein construct is functional.

Immunofluorescence staining revealed a barely detectable signal for endogenous SOX5 in GFP-transduced RAJI cells (Fig. S3B); whereas SOX5 was highly expressed and the co-expression and nuclear localization of exogenous SOX5 and GFP was readily documented in SOX5-GFP-transduced cells (Fig. S3C).

Subsequently, expression levels of previously reported SOX5 target genes *RhoB* [28], *S100A1* and *S100B* [29] were examined in sorted GFP-low and GFP-hi control cells and SOX5-GFP-low, SOX5-GFP-intermediate (int) and SOX5-GFP-hi cells (Fig. S3D). While *RHOB* and *S100B* genes seemed not to be expressed in RAJI cells (Fig. S3E), the *S100A1* gene was expressed and its expression levels correlated with the expression levels of *5′L3S*-*SOX5* in SOX5-GFP transduced RAJI cell fractions (Fig. S3D and S3E), compatible with the regulation of the *S100A1* gene by exogenous SOX5. Unlike in chondrocytes [29], the regulation of the *S100A1* did not depend on SOX-trio activity, since *SOX6* and *SOX9* were not expressed in RAJI cells (Fig. S3E).

**Figure 6:**
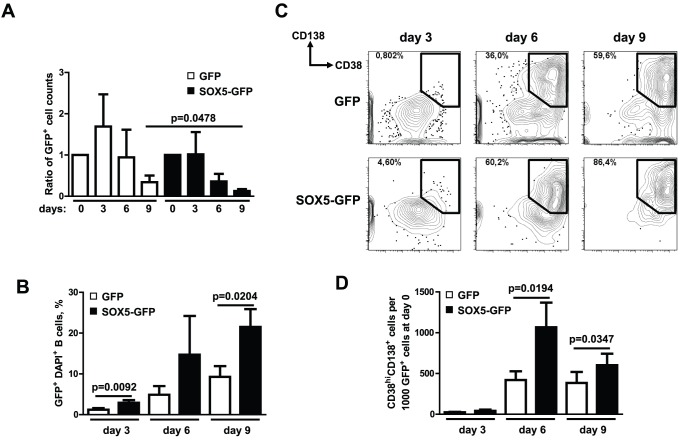
SOX5 modulates *in vitro* terminal B cell differentiation. (A) Proliferation measured as the ratio of absolute numbers of GFP^+^ cells in the samples. Absolute cell counts of GFP^+^ cells on day 0 are taken as 1.0 and the data are expressed as mean ± SD. Summary of three independent experiments are depicted. (B) Frequencies of GFP^+^ DAPI^+^ cells within GFP (control) and SOX5-GFP-transduced peripheral blood B cells cultivated *in vitro*. (C) Plasma cell differentiation analyzed by FACS in peripheral blood B cells stably transduced either with GFP (control) or SOX5-GFP fusion construct upon *in vitro* stimulation with IL4+CD40L+IL21. DAPI-negative GFP^+^ gated cells were analyzed by FACS for the plasma cell markers CD138 and CD38 at days 3, 6 and 9. Gates indicate the frequencies of CD138^+^CD38^hi^ plasmablasts in each FACS plot. Representative FACS plots of three independent experiments are shown. (D) Numbers of CD138^+^CD38^hi^ plasmablasts in GFP (control) and SOX5-GFP-transduced B cells at days 3, 6 and 9 referring to CD38^hi^ CD138^+^ cells per 1000 GFP^+^ cells at day 0. Cell numbers are depicted as mean ± SD and t-test p-values indicate the significant differences. Summary of three independent experiments are shown.

After confirmation of the expression and function of the fusion protein in a human B cell line, we analyzed the function of L-SOX5F in primary human B lymphocytes. Primary B lymphocytes were transduced with SOX5-GFP fusion and GFP control constructs. Isolated cells were stimulated for 9 days *in vitro* (Fig. 6). Analysis of absolute counts of GFP-positive cells at day 0 and at days 3, 6 and 9 showed that GFP-infected cells almost doubled on day 3 (ratio day 3/day 0 = 1.7±0.8; n = 3), while the absolute counts of SOX5-GFP-positive cells did not increase (ratio day 3/day 0 = 1.0±0.5; n = 3; Fig. 6A). In accordance with a reduced proliferative capacity of cells transduced with SOX5-GFP we found the up-regulation of a negative cell cycle regulator gene *CHEK1* and cyclin E1 (*CCNE1*) gene, whereas several positive cell cycle regulator genes, including *CCND1/2*, *CDK4*, *CDC25A*, *TFDP2*, *E2F4* and *MKI67*, were down-regulated in SOX5-GFP transduced B cells at day 3 compared to the GFP control (Fig. S4). DAPI staining of the cells revealed relatively increased frequencies of GFP^+^ DAPI^+^ dead cells upon SOX5-GFP transductions at day 3 (2.9% ±1.1%; n = 3; p = 0.0092), day 6 (14.7% ±16.5%; n = 3) and day 9 (21.5% ±7.6%; n = 3; p = 0.0204) compared to the GFP control transductions at day 3 (1.2% ±0.7%; n = 3), day 6 (4.9% ±3.7%; n = 3) and day 9 (9.3% ±4.6%; n = 3), respectively (Fig. 6B). Compatible with the increased apoptosis we saw the anti-apoptotic gene *BIRC5* down-regulated in SOX5-GFP transduced B cells (Fig. S4). The flow cytometric analysis of cultured B lymphocytes (Fig. 6C) revealed a relative, but not absolute expansion of CD138^+^CD38^hi^ plasmablasts (counted as numbers of CD38^hi^ CD138^+^ cells per 1000 GFP^+^ cells at day 0) in SOX5-GFP-transduced cells at day 6 (1067±741; n = 3; p = 0.0194) and day 9 (602±345; n = 3; p = 0.0347), when compared to the control GFP-transduced cells at days 6 (420±261; n = 3) and 9 (384±331; n = 3), respectively (Fig. 6D). These data corroborate the negative effect of SOX5 expression on proliferation, whereas plasmablast differentiation appears to be spared.

## Discussion

Here we report for the first time a detailed analysis of the *SOX5* expression in human B lymphocytes. Five different representative transcript variants of human *SOX5* are identified in the GenBank database. Among the known transcript variants of human *SOX5*, only the variants 2 and 3 are expressed in human B cells. By PCR cloning of the full CDS and subsequent sequence verification we identified two new variants of human *SOX5* transcripts, the variants 6 (5′L3L-SOX5) and 7 (5′L3S-SOX5), for which GenBank accession numbers JX570584 and JX570585 were assigned, respectively. Similar to the variant 2, both of the new transcript variants carry 18 exons, but bear an additional 75 bp non-coding exon 5. The two new *SOX5* transcript variants differ in the length of coding exon 3. In the transcript variant 6 the coding exon 3 (coding exon 3a) is present in its full length of 232 bp coding for 77 aa, whereas in the transcript variant 7 the coding exon 3 (coding exon 3b) is only 127 bp long sustaining the same translation initiation site, but coding for only 42 aa (Fig. 2A). Like transcript variant 2, the transcript variant 6 encodes the known 750 aa protein isoform - L-SOX5B (NP_694534.1), whereas the transcript variant 7 encodes a new, shorter, 715 aa long protein isoform of SOX5, which we designated L-SOX5F. The missing 35 aa residues in the L-SOX5F isoform do not involve any of the known functional domains of L-SOX5 (Fig. S5). Both new variants are expressed in nearly equal amounts in all tested tonsillar B cell populations and during B cell activation *in vitro,* suggesting similar regulation of expression due to identical regulatory elements. However, due to the low sensitivity and limitations of the fragment analysis technique applied in this study, we cannot exclude differential expression of the two SOX5 variants in some of the circulating B cells, especially in circulating CD21^low^ and atypical memory B cells.

SoxD proteins have no known transactivation or transrepression domains and therefore require interaction partners to participate in transcriptional activation and repression as demonstrated in different cell types [3], [4]. The SOX-trio, consisting of SOX5, SOX6 and SOX9 [26], is so far the best-examined complex involving the action of Sox5 in chondrocytes [8], [30], oligodendrocytes and melanocytes [4], [9], [31]. Depending on the constellation Sox5 and Sox6 heterodimers cooperate with Sox9 in transactivation of chondrocyte-specific genes [3], [4] or act as transrepressors by competing with SoxE proteins in oligodendrocytes and melanocytes by directly recruiting the co-repressor CtBP2 and the histone deacetylase Hdac1 [3], [4]. Beside the interaction of L-Sox5 and Sox6 proteins [32], co-expression of L-Sox5 and Sox13 was shown in mouse pancreatic epithelial cells [27]. However, our data clearly indicate that neither *SOX6*, *SOX9,* nor *SOX13* are expressed in circulating B cells nor induced after *in vitro* activation of human B lymphocytes. Therefore other interaction partners for SOX5 must exist in human B cells and point to a cell type-dependent expression of interaction partners for SOX5.

*SOX5* transcription varied between peripheral blood B-cell subpopulations, showing the highest expression levels in circulating innate-like CD21^low^ B cells and non-classical CD27^−^ memory B cell populations, as we had previously seen in CD21^low^ B cells of patients with CVID [16] and others in FCRL4^+^ tissue-like memory B cells of tonsils [15]. The comparison of tonsillar B cell populations in healthy donors revealed the highest expression levels of *SOX5* transcript in GC B cells as previously reported [15]. In tonsillar sections the expression of SOX5 protein was polarized to centrocytes within the light zones of germinal centers, but not in extrafollicular CD138^+^ plasma cells, suggesting that *SOX5* expression is upregulated during the GC response of B cells and switched off after terminal differentiation.

In order to dissect the expression pattern of *SOX5* during B cell activation, proliferation and differentiation more closely, we investigated the *SOX5* expression at different time points after stimulation of B cells *in vitro* with IL4, IL21, CD40L alone or a combination of these. The highest SOX5 transcript levels were found in cells stimulated with IL21 alone, while SOX5 transcript levels were significantly reduced in cells undergoing proliferation (CD40L+IL4). Interestingly, adding IL21 to this stimulation not only caused a dramatic increase in plasmablast differentiation but also a slight, but significant up-regulation of *SOX5* expression. Given the high expression of SOX5 protein in centrocytes in the light zone of tonsils, these data suggest that SOX5 plays a role during IL21-mediated differentiation of activated B cells. This role, however, is not restricted to IL21 activation, since SOX5 was also induced in B lymphocytes undergoing plasmablast differentiation upon CpG stimulation *in vitro*.

In order to demonstrate the effect of high *SOX5* expression levels during B cell activation further, the complete CDS of the prominently expressed transcript variant 7, encoding the L-SOX5F, was cloned together with a *GFP* gene into a lentiviral expression vector. After transduction of human primary B cells with *SOX5-GFP* we found less SOX5-GFP positive cells at days 3, 6 and 9 compared to *GFP* transduced controls, after adjusting for transduction efficiency. SOX5-GFP positive cells failed to expand on day three after stimulation. In support of a negative influence of high SOX5 levels on the cell cycle we observed an up-regulation of negative regulators of the cell cycle in combination with the down-regulation of positive regulatory genes in the SOX5-GFP transduced cells. The increased expression of a negative regulator *CHEK1* and especially *CCNE1* is suggestive of a block at the G1-S phase transition [33]. This is consistent with the previous results in SOX5 expressing FCRL4 B cells, which don’t proliferate [34] and are mostly in G1 phase [15]. Similarly, in CD21^low^ B cells of CVID patients, the high level of *SOX5* expression is associated with poor proliferation, while a residual potential to differentiate into antibody secreting cells persists [16], [35]. There is multiple evidence for an anti-proliferative effect of SOX5 in non-B cells. Thus in neuronal progenitor cells high SOX5 expression is associated with a premature cell cycle exit [36] and in human glioma cells [37] with a poor proliferative capacity. Also the high expression of Sox5 in chondrocytes [8] is compatible with its role in the non-proliferative state of cells. A recent study corroborates that lentiviral overexpression of murine Sox5 proteins (Sox5-BLM and L-Sox5) inhibits cell cycle progression and results in increased apoptosis of human multiple myeloma cells [38]. We also observed an increased apoptosis rate of GFP^+^DAPI^+^ B cells upon transduction with SOX5-GFP. However, in contrast to the reported results, in our hands other B cells underwent apoptosis more readily and the percentage of CD138^+^CD38^hi^ plasmablasts accumulated with time significantly more in SOX5-GFP transduced B cells compared to GFP transduced B cells suggesting that plasma cells might be less strongly affected by high SOX5 expression levels. This difference might be reconciled by the fact, that a multiple myeloma line reflects rather terminally differentiated plasma cells which in deed express only very low levels of SOX5, while according to our *in vitro* results SOX5 is naturally up-regulated during the process of plasma cell differentiation.

In conclusion, we reported here the identification of two new transcript variants of human *SOX5*, variant 6 and variant 7, which are expressed in B cells and encode the known 750 aa L-SOXB isoform and a new isoform of 715 aa L-SOX5F, respectively. SOX5 expression is stage dependent during B cell differentiation. It shows the highest expression in centrocytes in the light zones of germinal centers, in circulating atypical memory and exhausted CD21^low^ B cells. The interaction partners in B cells remain unknown but functional assays suggest that it is induced by IL21 and that high levels of L-SOX5F modify terminal B cell differentiation by suppressing proliferation, but permitting plasmablast development.

## Supporting Information

Figure S1 **Expression of 5′L3L-SOX5 and 5′L3S-SOX5 transcripts in human B cell subpopulations.** (A) A scheme for primer locations and (B) relative quantification of 5′L3L-SOX5 by RT-qPCR in peripheral blood naive, MZ-like, switched memory (sw mem), non-classical memory (nc mem) and CD21low B cells as well as (C) in follicular naive, germinal center B cells (GC) and memory B cells from tonsils. T-test p-values indicate the significance of differences between the samples. Relative expression levels of 5′L3L-SOX5 are shown as mean ± SD. RPLP0 gene was used as an internal control in the samples. Fragment analysis for the relative expression of 5′L3S-SOX5 transcript (D) in tonsillar B cell populations and (E) in CD19+ peripheral blood B cells upon stimulation in vitro at days 3, 6 and 9. The cells were activated either with a single stimulus or with a combination of stimuli, as indicated in the figure (E).
(PDF)

Figure S2 **Expression of SOX5 transcripts upon CpG-mediated B cell differentiation in vitro.** (A) Differentiation of B cells upon stimulation with CpG in vitro. The gates depict CD138+CD38hi plasmablasts at days 3, 6 and 9. (B) RT-qPCR analysis of SOX5 expression in samples stimulated with CpG. T-test p-values indicate the significance of differences between the samples. Relative expression levels of SOX5 are shown as mean ± SD. RPLP0 gene served as an internal control in the samples.
(PDF)

Figure S3 **Construction of the SOX5-GFP fusion protein and its functionality upon lentiviral transduction in RAJI cells.** (A) Luciferase promoter reporter assays for GFP-control and SOX5-GFP fusion constructs in BEAS-2B cells. Stably transduced BEAS-2B cells either expressing GFP alone or SOX5-GFP fusion protein were GFP-sorted and subsequently transient transfection was performed to measure the promoter activity. pGL3-Basic plasmid was used as a control for human SPAG6 promoter constructs, pGL3-1-SOX5 and pGL3-4-SOX5. Appropriate t-test p-values indicate the significance of differences between GFP control and SOX5-GFP expressing cells. (B) and (C) Immunofluorescence staining for SOX5 protein in RAJI cells. RAJI cells were transduced either with GFP control vector (B) or SOX5-GFP fusion construct (C). Co-localization of GFP (green) and SOX5 (red – TRITC) and nuclear translocation is shown. DAPI staining is indicative of cellular nuclei. (D) Lentiviral expression of GFP and SOX5-GFP fusion proteins in RAJI cells analyzed by flow cytometry. Stably transduced RAJI cells were sorted into GFP-low and GFP-hi as well as SOX5-GFP-low, SOX5-GFP-int and SOX5-GFP-hi fraction and RT-PCR analyses for the expression of GAPDH and 5′L3S-SOX5 transcript were performed. (E) RT-PCR analysis for the expression of known SOX5 target genes: RHOB, S100A1 and S100B as well as SOX-trio genes, SOX6 and SOX9 in stably transduced and GFP-sorted RAJI cell fractions. In agarose gel pictures DNA markers were cut out, since they were loaded between the tested samples and the control sample. Human costal cartilage cells served as a control.
(PDF)

Figure S4 **Expression of human cell cycle genes in SOX5-transduced and** ***in vitro*** **differentiated human primary B cells.** Human cell cycle RT2 Profiler PCR Array containg 84 pathway specific genes were run for RNA samples of GFP- and SOX5-GFP-transduced human primary B cells at day 3 upon CD40L+IL4+IL21 stimulation in vitro. Negative regulatory genes of cell cycle are indicated in red, whereas positive regulatory genes of cell cycle are depicted in green. Black color shows the regulation of RAD51 gene, which is involved in the homologous recombination and repair of DNA. Dashed lines indicate a threshold of 2 fold up- or down-regulation of genes.
(PDF)

Figure S5 **Comparison of known functional domains in L-SOX5B and L-SOX5F proteins.** Both SOX5 proteins contain the same known functional domains and share the same translation initiation site, except for different exon 3 (exon 3a and exon 3b). NL – nuclear localization domain.
(PDF)

Table S1 **Primer sequences used in this study.** Primer sequences are listed and subdivided into sections according to the assay type and in the order as they appear in the text.
(PDF)
