# Supplementary figures and images for: High Levels of SOX5 Decrease Proliferative Capacity of Human B Cells, but Permit Plasmablast Differentiation
Source: PLoS One. 2014 Jun 19;9(6):e100328. doi: 10.1371/journal.pone.0100328 (PMC4063782; doi:10.1371/journal.pone.0100328)

**A**SOX5 transcriptSOX5 protein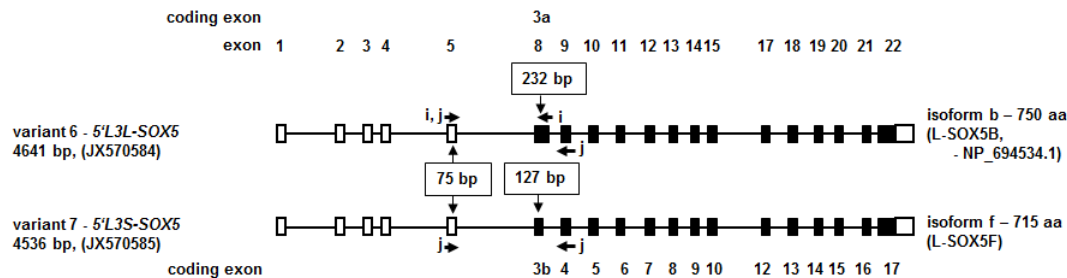**B**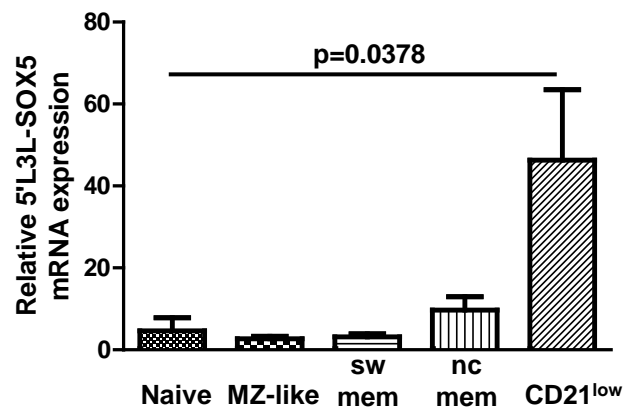**C**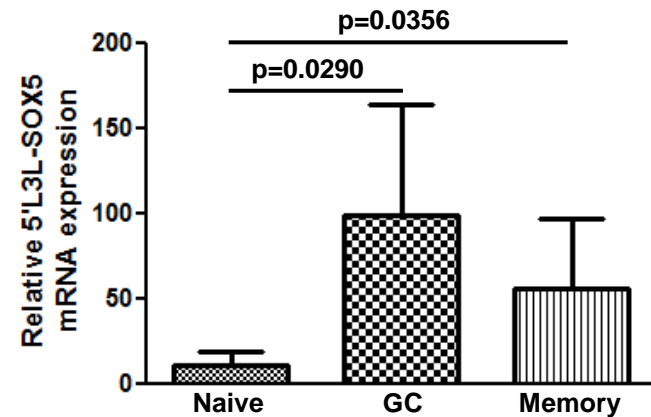**D**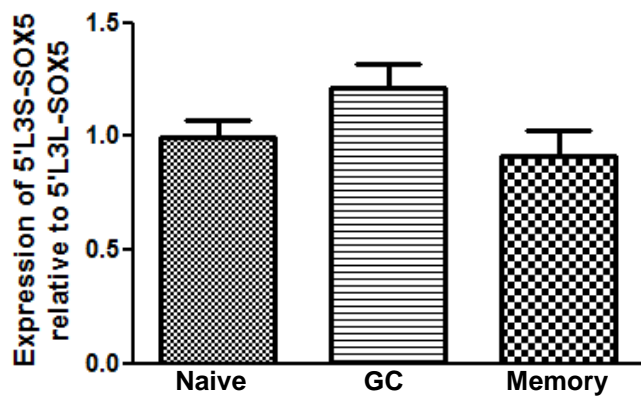**E**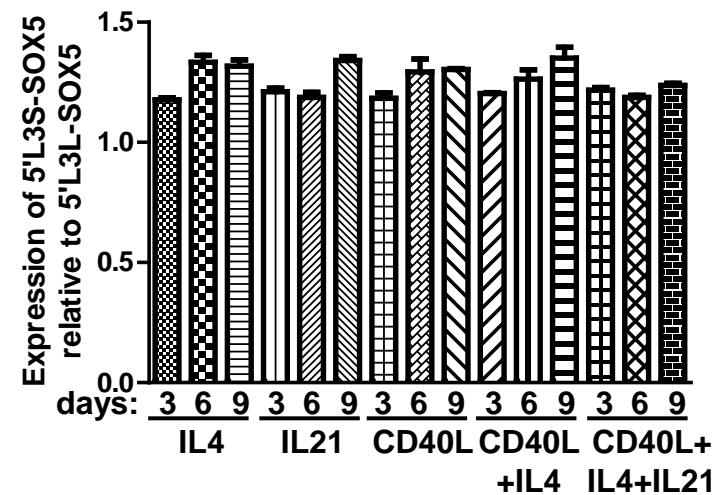

Figure S1.

Supplement: Figure S1 — Expression of 5′L3L-SOX5 and 5′L3S-SOX5 transcripts in human B cell subpopulations. (A) A scheme for primer locations and (B) relative quantification of 5′L3L-SOX5 by RT-qPCR in peripheral blood naive, MZ-like, switched memory (sw mem), non-classical memory (nc mem) and CD21low B cells as well as (C) in follicular naive, germinal center B cells (GC) and memory B cells from tonsils. T-test p-values indicate the significance of differences between the samples. Relative expression levels of 5′L3L-SOX5 are shown as mean ± SD. RPLP0 gene was used as an internal control in the samples. Fragment analysis for the relative expression of 5′L3S-SOX5 transcript (D) in tonsillar B cell populations and (E) in CD19+ peripheral blood B cells upon stimulation in vitro at days 3, 6 and 9. The cells were activated either with a single stimulus or with a combination of stimuli, as indicated in the figure (E). (PDF) [file pone.0100328.s001.pdf]

**A**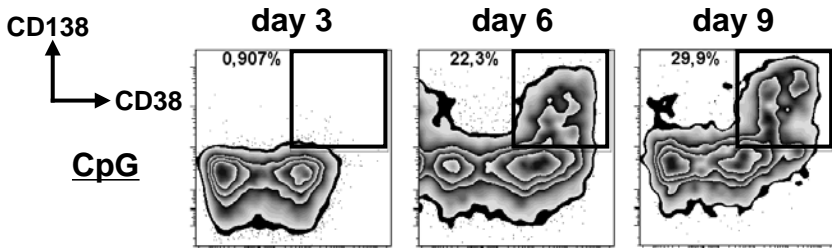**B**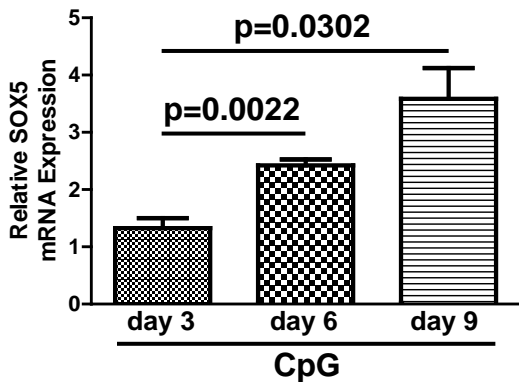

Figure S2.

Supplement: Figure S2 — Expression of SOX5 transcripts upon CpG-mediated B cell differentiation in vitro. (A) Differentiation of B cells upon stimulation with CpG in vitro. The gates depict CD138+CD38hi plasmablasts at days 3, 6 and 9. (B) RT-qPCR analysis of SOX5 expression in samples stimulated with CpG. T-test p-values indicate the significance of differences between the samples. Relative expression levels of SOX5 are shown as mean ± SD. RPLP0 gene served as an internal control in the samples. (PDF) [file pone.0100328.s002.pdf]

**A**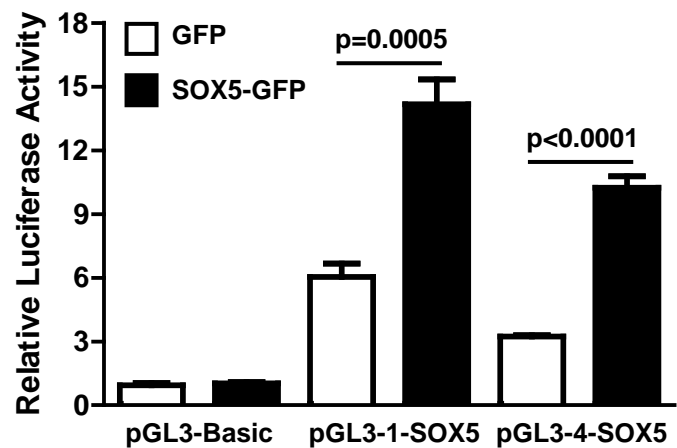**D**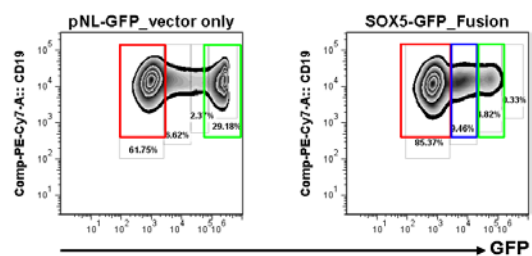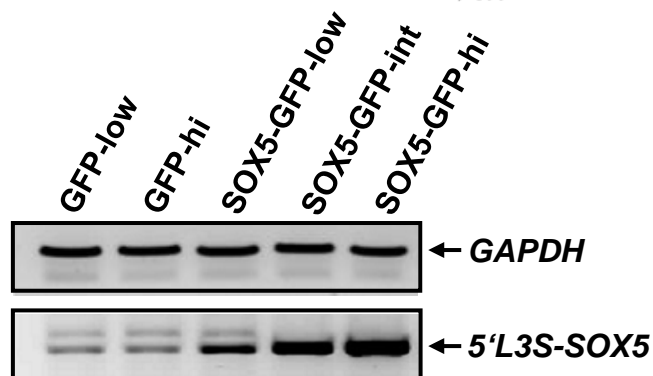**B**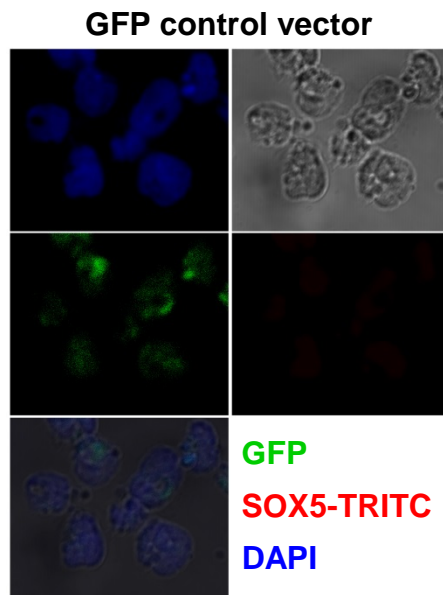**C**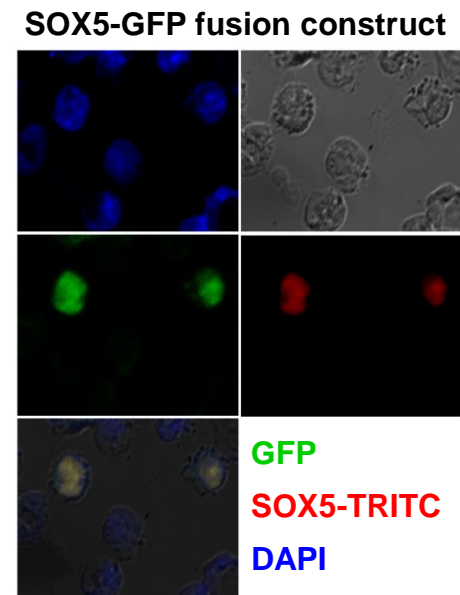**E**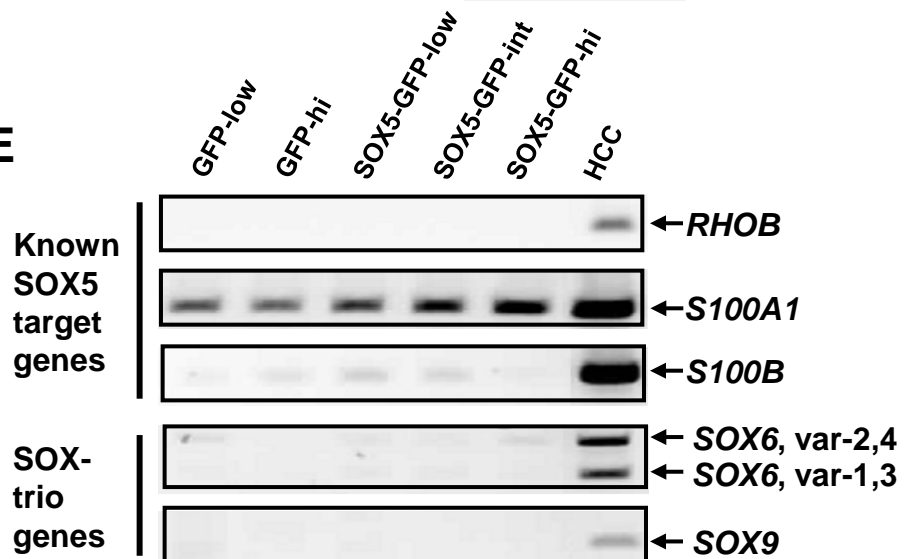

Figure S3.

Supplement: Figure S3 — Construction of the SOX5-GFP fusion protein and its functionality upon lentiviral transduction in RAJI cells. (A) Luciferase promoter reporter assays for GFP-control and SOX5-GFP fusion constructs in BEAS-2B cells. Stably transduced BEAS-2B cells either expressing GFP alone or SOX5-GFP fusion protein were GFP-sorted and subsequently transient transfection was performed to measure the promoter activity. pGL3-Basic plasmid was used as a control for human SPAG6 promoter constructs, pGL3-1-SOX5 and pGL3-4-SOX5. Appropriate t-test p-values indicate the significance of differences between GFP control and SOX5-GFP expressing cells. (B) and (C) Immunofluorescence staining for SOX5 protein in RAJI cells. RAJI cells were transduced either with GFP control vector (B) or SOX5-GFP fusion construct (C). Co-localization of GFP (green) and SOX5 (red – TRITC) and nuclear translocation is shown. DAPI staining is indicative of cellular nuclei. (D) Lentiviral expression of GFP and SOX5-GFP fusion proteins in RAJI cells analyzed by flow cytometry. Stably transduced RAJI cells were sorted into GFP-low and GFP-hi as well as SOX5-GFP-low, SOX5-GFP-int and SOX5-GFP-hi fraction and RT-PCR analyses for the expression of GAPDH and 5′L3S-SOX5 transcript were performed. (E) RT-PCR analysis for the expression of known SOX5 target genes: RHOB, S100A1 and S100B as well as SOX-trio genes, SOX6 and SOX9 in stably transduced and GFP-sorted RAJI cell fractions. In agarose gel pictures DNA markers were cut out, since they were loaded between the tested samples and the control sample. Human costal cartilage cells served as a control. (PDF) [file pone.0100328.s003.pdf]

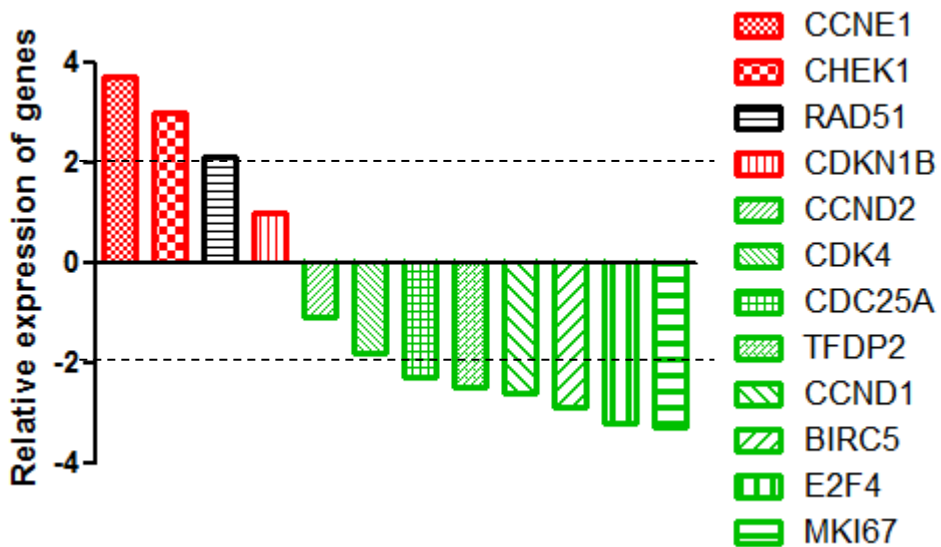

Figure S4.

Supplement: Figure S4 — Expression of human cell cycle genes in SOX5-transduced and in vitro differentiated human primary B cells. Human cell cycle RT2 Profiler PCR Array containg 84 pathway specific genes were run for RNA samples of GFP- and SOX5-GFP-transduced human primary B cells at day 3 upon CD40L+IL4+IL21 stimulation in vitro. Negative regulatory genes of cell cycle are indicated in red, whereas positive regulatory genes of cell cycle are depicted in green. Black color shows the regulation of RAD51 gene, which is involved in the homologous recombination and repair of DNA. Dashed lines indicate a threshold of 2 fold up- or down-regulation of genes. (PDF) [file pone.0100328.s004.pdf]

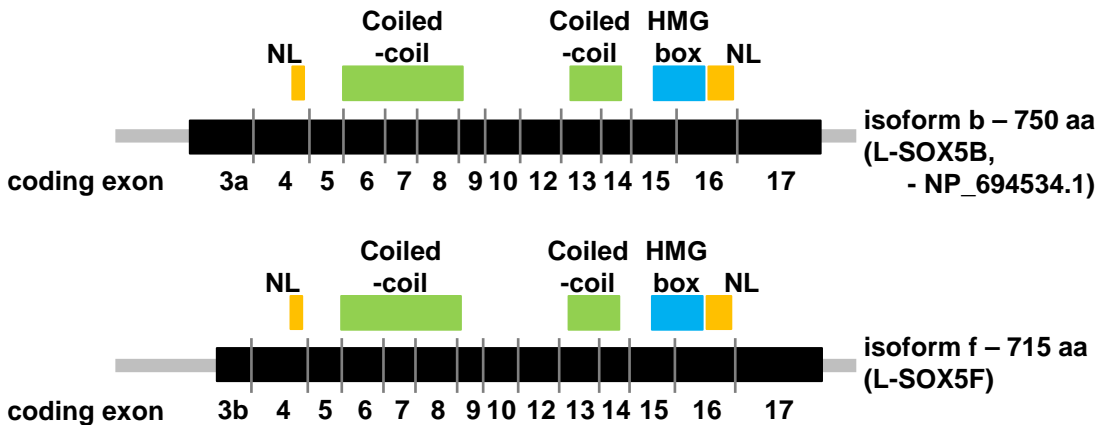

**Figure S5.**

Supplement: Figure S5 — Comparison of known functional domains in L-SOX5B and L-SOX5F proteins. Both SOX5 proteins contain the same known functional domains and share the same translation initiation site, except for different exon 3 (exon 3a and exon 3b). NL – nuclear localization domain. (PDF) [file pone.0100328.s005.pdf]
