# Supplementary material for: High Levels of SOX5 Decrease Proliferative Capacity of Human B Cells, but Permit Plasmablast Differentiation
Source: PLoS One. 2014 Jun 19;9(6):e100328. doi: 10.1371/journal.pone.0100328 (PMC4063782; doi:10.1371/journal.pone.0100328)
Supplement: Table S1 — Primer sequences used in this study. Primer sequences are listed and subdivided into sections according to the assay type and in the order as they appear in the text. (PDF) [file pone.0100328.s006.pdf]

Table S1. Primer sequences used in this study.

SOX5 PCR primer pair	Primer name	Primer sequence
	**PCR primers:**	
	β-actin forward	5'-ATC ATT GCT CCT CCT GAG CG-3'
	β-actin reverse	5'-TCA CCT CCC CTG TGT GGA CT-3'
	CD19 forward	5'-CGG GAG TGG GCC CAG AAG AAG AGG-3'
	CD19 reverse	5'-CCA GGC TGG CCC CGA ATG GAG-3'
a	SOX5-var-1 forward	5'-AGC ATG CTT ACT GAC CCT GAT TTA-3'
a	SOX5-var-1 reverse	5'-GGG AGT CCT ATG GCC ACA AGT CT-3'
b	SOX5-var-5 forward	5'-CAC GGG GCA GCC ACC ATC T-3'
b	SOX5-var-5 reverse	5'-CGC CCA CCT TCT TCT GCC TTC T-3'
c	SOX5-var-3 forward	5'-TTG TGC CAT AGG AGC TGT GCA T-3'
c	SOX5-var-3 reverse	5'-TTC GGC AGT TAT TGA GAC CCA GAC-3'
d	SOX5-var-2/4 forward (5'-end)	5'-GCG ACC TAC TGG GCT TTC CTC TTG-3'
d	SOX5-var-2/4 reverse (5'-end)	5'-CCG TCA CTC TCC TCT TCT TCC ACT-3'
e	SOX5-var-2/4 forward (exon-15)	5'-AAC CTT GGT GCT GCT GTA TCT CCT-3'
e	SOX5-var-2/4 reverse (exon-20)	5'-CCG TTC ATC TTT AGC CCA CAC CAT-3'
f	SOX5-exon-22 forward	5'-CAA GCG TGT CTA GCA GCC C-3'
f	SOX5-exon-22 reverse	5'-TGG CTT GTC CTG CAA TAT GGT-3'
g	SOX5-CDS forward (exon-3/4)	5'-GCG ACC TAC TGG GCT TTC CTC TTG-3'
g	SOX5-CDS reverse (exon-22)	5'-CTC TTT TTC CCT CTT TCC CCT CAT-3'
h	SOX5-exon-3/4 forward	5'-GCG ACC TAC TGG GCT TTC CTC TTG-3'
h	SOX5-exon-9 reverse (coding exon-4)	5'-TCG CCC ACC TTC TTC TGC CTT CTG-3'
	RPLP0 forward	5'-CGG GCC CGA GAA GAC CT-3'
	RPLP0 reverse	5'-TTG TAC CCG TTG ATG ATA GAA TGG-3'
	SOX6 forward	5'-TGA TCC CAA TTT TTC CAC AT-3'
	SOX6 reverse	5'-CCA GCT GAG CGG CAT AGA-3'
	SOX9 forward	5'-GCG GCG GAG GAA GTC GGT GAA GA-3'
	SOX9 reverse	5'-ACG TCG GTT TTG GGG GTG GTG-3'
	SOX13 forward	5'-GCC ATA GAG AAG CTG TTG TCC-3'
	SOX13 reverse	5'-ATC TTA TGC TGC TGC TGA ATC-3'
	GAPDH forward	5'-GAA GGT GAA GGT CGG AGT C-3'
	GAPDH reverse	5'-GAA GAT GGT GAT GGG ATT TC-3'
h	5'L3S-SOX5 forward (exon-3/4)	5'-GCG ACC TAC TGG GCT TTC CTC TTG-3'
h	5'L3S-SOX5 reverse (exon-9/coding exon-4)	5'-TCG CCC ACC TTC TTC TGC CTT CTG-3'
	RHOB forward	5'-GGC CAT CCG CAA GAA GC-3'
	RHOB reverse	5'-CGC AGG CGG TCG TAG TCC-3'
	S100A1 forward	5'-TCG GGC AAA GAG GGG GAC AAG TA-3'
	S100A1 reverse	5'-TGA GAG CAG CCA CAA GCA CCA CA-3'
	S100B forward	5'-GCC CTC ATC GAC GTT TTC CAC CAA-3'
	S100B reverse	5'-AGT CAC ATT CGC CGT CTC CAT CAT-3'
	**Real time qPCR primers:**	
	RPLP0 forward	5'-CGG GCC CGA GAA GAC CT-3'
	RPLP0 reverse	5'-TTG TAC CCG TTG ATG ATA GAA TGG-3'
a	SOX5-exon-22 forward	5'-CAA GCG TGT CTA GCA GCC C-3'
a	SOX5-exon-22 reverse	5'-TGG CTT GTC CTG CAA TAT GGT-3'
	BLIMP1 forward	5'-GAC TTT GCA GAA AGG CTT CAC T-3'
	BLIMP1 reverse	5'-ACA TTC TTT GGG CAG AGT TCA T-3'
i	5'L3L-SOX5 forward	5'-AGA GAT TGG ACT GGG AGA C-3'
i	5'L3L-SOX5 reverse	5'-CAT TGT ATT GTG CTG AGA AGT G-3'

Table S1. (continued).

SOX5 PCR primer pair	Primer name	Primer sequence
	**CDS cloning and sequencing primers:**	
g	SOX5-CDS forward (exon-3/4)	5’-GCG ACC TAC TGG GCT TTC CTC TTG-3’
g	SOX5-CDS reverse (exon-22)	5’-CTC TTT TTC CCT CTT TCC CCT CAT-3’
	M13 forward	5’-GTA AAA CGA CGG CCA GT-3’
	M13 reverse	5’-GGA AAC AGC TAT GAC CAT G-3’
	SOX5-CDS-seq forward-1	5’-ACG GCG CAA GGG CAG TTT AG-3’
	SOX5-CDS-seq reverse-1	5’-GAG GTG GGT GAT GTG GGT GAT TTG-3’
	SOX5-CDS-seq forward-2	5’-AAC CTT GGT GCT GCT GTA TCT CCT-3’
	SOX5-CDS-seq reverse-2	5’-CCG TTC ATC TTT AGC CCA CAC CAT-3’
	SOX5-CDS-seq forward-3	5’-GGG AAG GTG GCT GTT GTG AAT AGT-3’
	SOX5-CDS-seq reverse-3	5’-AGG TGC TGC TTG CTG AGA CG-3’
	SOX5-CDS-seq forward-4	5’-TGT GGG CTA AAG ATG AAC GGA GAA-3’
	SOX5-CDS-seq reverse-4	5’-CTG GGC TGC TAG ACA CGC TTG AG-3’
	SOX5-CDS-seq forward-5	5’-TGG GCA ACA AGC ACA GAT-3’
	SOX5-CDS-seq reverse-5	5’-AAA AAG TTG ACG GGT AAG AC-3’
	**Primers for fragment analysis**	
j	5’-FAM-SOX5-5’L forward	5’-5AG AGA TTG GAC TGG GAG AC-3’
j	5’-FAM-SOX5 reverse (coding exon-4)	5’-5TC GCC CAC CTT CTT CTG CCT TCT G-3’
	**Primers for SOX5-GFP fusion construct:**	
	EcoRI-SOX5 forward	5’-TGC CAG AAT TCC ACT GCA CTG GGT AGG AA-3’
	BamHI-SOX5 reverse	5’-ACC GGT GGA TCC CAG TTG GCT TGT CCT GCA-3’
